# Influence of the distensibility of large arteries on the longitudinal impedance: application for the development of non-invasive techniques to the diagnosis of arterial diseases

**DOI:** 10.1186/1753-4631-6-2

**Published:** 2012-04-16

**Authors:** Wassila Sahtout, Ridha Ben Salah

**Affiliations:** 1 Biomedical Engineering Department, Higher Institute of Biotechnology of Sfax, Route de la Soukra BP261, 3032, Sfax, Tunisie; 2 Unit of research of Biophysics, Faculty of Medicine of Sousse, Sousse, Tunisie

## Abstract

**Background:**

This study shows that the arterial longitudinal impedance constitutes a hemodynamic parameter of interest for performance characterization of large arteries in normal condition as well as in pathological situations. For this purpose, we solved the Navier–Stokes equations for an incompressible flow using the finite element analysis method and the Arbitrary Lagrangian Eulerian (ALE) formulation. The mathematical model assumes a two-dimensional flow and takes into account the nonlinear terms in the equations of fluid motion that express the convective acceleration, as well as the nonlinear deformation of the arterial wall. Several numerical simulations of the blood flow in large vessels have been performed to study the propagation along an arterial vessel of a pressure gradient pulse and a rate flow pulse. These simulations include various deformations of the wall artery leading to parietal displacements ranging from 0 (rigid wall) to 15% (very elastic wall) in order to consider physiological and pathological cases.

**Results:**

The results show significant changes of the rate flow and the pressure gradient wave as a function of aosc, the relative variation in the radius of the artery over a cardiac cycle. These changes are notable beyond a critical value of aosc equal to 0.05. This critical value is also found in the evolution of the longitudinal impedance. So, above a variation of radius of 5%, the convective acceleration, created by the fluid-wall interactions, have an influence on the flow detectable on the longitudinal impedance.

**Conclusions:**

The interpretation of the evolution of the longitudinal impedance shows that it could be a mean to test the performance of large arteries and can contribute to the diagnosis of parietal lesions of large arteries. For a blood vessel with a wall displacement higher than 5% similar to those of large arteries like the aorta, the longitudinal impedance is substantially greater than that obtained in the absence of wall displacement. This study also explains the effects of convective acceleration, on the shape of the decline of the pressure gradient wave and shows that they should not be neglected when the variation in radius is greater than 5%.

## Background

The elasticity of large arteries which represent the principal arterial function made that they constitute a very distensible reservoir. Their role is to transform pulsatile flow at the outlet of the heart in a continuous flow in the capillary bed. Consequently, the loss of distensibility is considered a cardiovascular risk factor [1]. This increased rigidity is due to age [2-4] or to various diseases such as hypertension or atherosclerosis [5-8]. Several methods have been proposed for measuring the elasticity of arteries, these methods are based on several techniques such that tonometry, mechanical transducers, echotracking, ultrasonic Doppler, functional Magnetic Resonance Imaging (MRI) and photoplethysmography [9]. However, these methodologies have limitations because some of them may apply only to superficial arteries. Others, such that ultrasonic Doppler and functional MRI, where the site of interest is the deep arteries, are mainly based on the linear theory developed by Womersley [10,11], which is not applicable in the case of large arteries like the aorta. Indeed, the wall displacements of the large arteries are of finite amplitude and thus the convective acceleration (advection terms) introduce important nonlinear effects [12-14]. In normal physiological situations, the change in radius of these arteries is greater than 10% of the diastolic radius; this is due to the large number of elastin fibers, highly elastic, despite the presence of collagen fibers in the arterial wall. The collagen fibers having an elastic modulus of 3 to 100 times greater than that of elastin fibers are solicited from a pressure of 120mmHg [15,16]. Beyond that pressure, and especially from 140mmHg (hypertension), we observe a hardening of the arteries and a nonlinear mechanical behavior of the wall.

In this study we are studying the effect of large deformations induced by the mechanical behavior of large arteries (radius of about 1 cm) on the longitudinal impedance. The longitudinal impedance would be an index of clinical interest to quantify the performance of local arterial function especially if it is measured using the techniques of Doppler and MRI. For this purpose, we have solved numerically the equation of Navier–Stokes using Finite Element Analysis (FEA), taking into account the fluid - structure interactions expressed by the convective acceleration terms. To be in a real situation, i.e. in physiologically conditions normal and pathological, we determined the hemodynamic quantities in the arteries where the radius varies from 0% (rigid arterial wall) up to 15% (arterial wall very elastic) of the diastolic radius.

## Methods

For large arteries, blood can be considered Newtonian [17]. We have therefore considered it as a viscous incompressible fluid of dynamic viscosity η = 0.005 Pl and of density ρ = 1050 kg.m^-3^, flowing, in a cylindrical duct of diastolic radius set to 1 cm. The flow regime is characterized by the frequency parameter α introduced by Womersley which is equal to 11.49; this parameter reflects the importance of inertial effects relative to viscous effects. If the frequency pulsation is ω = 6.28 s^-1^, α is expressed as follows:

(1) $$\alpha =R_{0}\sqrt{\frac{\rho \omega }{\eta }}$$

### Governing equations

The mathematical model is based on axisymmetric flow described by the vector equation of Navier - Stokes and the continuity equation for a velocity field at time t, $\vec{V}\left[U\left(r,z,t\right),W\left(r,z,t\right)\right]$ and of pressure P (z, t) in the cylindrical coordinate system (r, z), where r and z are respectively the radial and axial coordinate. Knowing that the pressure is quasi-constant over the cross-sectional of the blood vessel area [18]

(2) $$\frac{\partial P}{\partial r}=0,\text{we get}:$$

(3) $$\frac{\partial U}{\partial r}+\frac{U}{r}+\frac{\partial W}{\partial z}=0\;$$

(4) $$\frac{\partial U}{\partial t}+U\frac{\partial U}{\partial r}+W\frac{\partial U}{\partial z}=\frac{\eta }{\rho }\left(\frac{\partial^{2}U}{\partial r^{2}}+\frac{1}{r}\frac{\partial U}{\partial r}+\frac{\partial^{2}U}{\partial z^{2}}-\frac{U}{r^{2}}\right)$$

(5) ∂W∂t+U∂W∂r+W∂W∂z=−1ρ∂P∂z+ηρ∂2W∂r2+1r∂W∂r+∂2W∂z2

Where W (r, z, t) and U (r, z, t) are the components of blood velocity in the longitudinal and radial directions and P (r, z, t) is the pressure. As we can see, equations (3) and (4) have nonlinear terms, corresponding to the convective accelerations. We have solved numerically these equations taking into account the nonlinear terms in order to study the influence of the distensibility on the flow. The nonlinear behavior of the arterial wall will be taken into consideration in the boundary conditions as described thereafter.

### Boundary conditions

The boundary conditions at the wall and at the center assume an axisymmetric flow and no-slip on the wall expressed as follow [18-20]:

(6) $$U(R,z,t)=\frac{\partial R}{\partial t}\;W(R,z,t)=0\;U(0,z,t)=0\;{\frac{\partial W}{\partial r}|}_{r=0}=0\;$$

R (z, t) is the inner radius of the vessel, which is a function of z and t because of the radial deformability. The first condition expresses that longitudinal movements of the arterial wall are neglected, which is largely justified in the work of Carew [17] and Patel [18]. The other boundary conditions at the wall (r = R) and at the center (r = 0) assume an axisymmetric flow and no slip on the wall.

We express the radial displacement of the arterial wall as follows:

(7) $$R\left(z,t\right)=R_{0}\times aosc\left[1-\cos\left(\omega t-\omega \frac{z}{c}\right)\right]+R_{0}$$

Where ΔR represents the amplitude of the radial displacement during a cardiac cycle and $aosc=\frac{\Delta R}{R_{0}}$ is the relative variation of the radius. To show the effect of distensibility of these arteries on the flow in normal and pathological conditions, we performed numerical simulations for different values of aosc ranging from 0 (rigid wall–severe pathological case) to 0.15 (very elastic wall). The phase velocity c was calculated from the relationship established by Thomas Young:

(8) $$c=\sqrt{\frac{R_{0}}{\rho }\frac{\Delta P}{\Delta R}}$$

ΔP is the amplitude of pressure corresponding to ΔR and so aosc. In large arteries the relationship between ΔP and ΔR is nonlinear due to the nonlinear dependence of the elastic modulus of arterial wall with the pressure [21]. The nonlinear behavior of the arterial wall is produced by imposing, at the entrance (z = 0), a sinusoidal pressure with a frequency of 1 Hz, in order to obtain an input rate flow virtually identical whatever the value of aosc:

(9) $$P(z=0)=\Delta P\left[1-\cos\left(\omega t\right)\right]+\bar{P}$$

The values of ΔP and of the average pressure $\overset{}{P}$ are set to yield a constant flow at z = 0. Thus, the nonlinear mechanical behavior is introduced in the terms ΔP and ΔP/ΔR.

### Numerical simulation

The simulation was performed using the finite element method and a formulation specifically Arbitrary Lagrangian Eulerian (ALE) [22], to take into account the fluid - structure interactions. In the fluid domain, a mixed formulation velocity - pressure has been implemented. ALE-methods are frequently used to model systems where the physical domain changes with respect to time. Common examples can be found in the field of fluid–structure interactions where the domain movement is due to the force that the fluid exerts on a solid object. These systems have gained a lot of interest to describe blood flows in hemodynamic.

**Figure 1:**
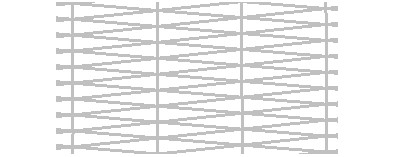
Sample of meshes used.

The calculations were performed using a 2D axisymmetric mesh of 26 elements in the radial direction and 250 elements in the axial direction for a length equal to 25cm (see Figure 1). We run the simulation on a Matlab platform for six values of aosc. Secondly, we have performed systematically a Fourier analysis of the pulses computed in our simulation (pressure, rate flow and pressure gradient). There are two approaches to explain the fluctuations of hemodynamic quantities. The first is to analyze the arterial pulse in the frequency domain through the description of the pulse in terms of its harmonic components (amplitude, frequency…). The second, traditional approach used in medical practice, is an analysis in the time domain to look for an explanation of the fluctuations of the arterial wave contour. We have chosen to apply the two approaches to correlate the shape of the wave-contour with the amplitude of different harmonics that compose it when the vascular distensibility varies.

The longitudinal impedance is calculated from the ratios between the amplitudes of pressure gradient $\frac{\partial P}{\partial z}$$\left[\frac{\partial \bar{P}}{\partial z},{\left(\frac{\partial \tilde{P}}{\partial z}\right)}_{1},{\left(\frac{\partial \tilde{P}}{\partial z}\right)}_{2}\right]$ and the rate flow Q $\left[\bar{Q},{\tilde{Q}}_{1},{\tilde{Q}}_{2}\right]$ obtained after analyze of Fourier. The expressions of the mean component, of the fundamental and second harmonic components of the longitudinal impedance are respectively:$\bar{Z}=\left|\frac{\frac{\partial \bar{P}}{\partial z}}{\bar{Q}}\right|$, ${\tilde{Z}}_{1}=\left|\frac{{\left(\frac{\partial \tilde{P}}{\partial z}\right)}_{1}}{{\tilde{Q}}_{1}}\right|$ and ${\tilde{Z}}_{2}=\left|\frac{{\left(\frac{\partial \tilde{P}}{\partial z}\right)}_{2}}{{\tilde{Q}}_{2}}\right|$.. We like to point out here that there is no linear relationship between the components of the pressure gradient and those of the flow since these quantities are the solutions of the nonlinear equations governing the flow. However, the Fourier analysis is quite justified by the fact that pressure gradient and rate flow are periodic signals.

## Results

### Input data

**Figure 2:**
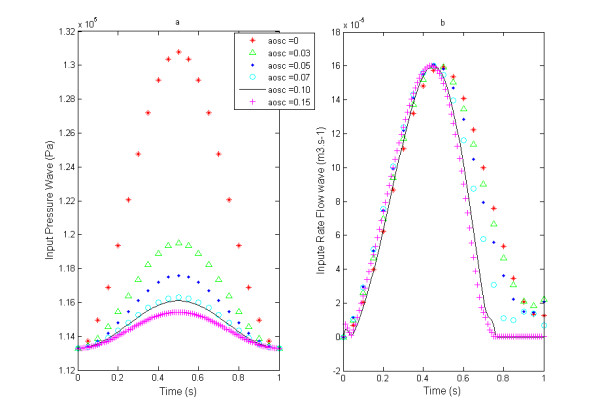
(a) Input signal of pressure – (b) Input signal of rate flow.

**Figure 3:**
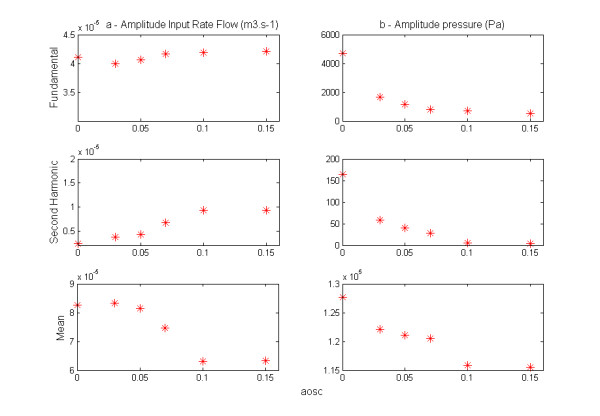
**(a) Amplitude of Input pressure – (b) Amplitude of Input of rate flow.** The amplitudes are obtained after Fourier transform implemented on the platform Matlab.

For each model (for each value of aosc) several tests were conducted in order to find the ideal pressure (time-dependant pressure) at the entrance of the vessel (z = 0) (Figures 2(a) and 3(a)), corresponding to a typical aortic waveform flow (z =0). The rate flow varies from 0 to 16.10^-5^m^3^. s^-1^ over the cardiac cycle, as shown in Figures 2(b) and 3(b). We observe in Figure 3(a) and (b) their amplitudes obtained after a Fourier analysis. To get the same rate flow at the entrance (z = 0) in each model, we see that the amplitudes of pressure we are selected decrease with aosc (Figure 2(a)). The wave of the rate flow (Figure 2(b)) has a distorted shape whereas the pressure wave at the entrance has been imposed as sinusoidal. Indeed, the Fourier analysis shows in Figure 3(b) the presence of a second harmonic whose amplitude becomes relatively large for aosc ≥ 0.07.

### Hemodynamic results

**Figure 4:**
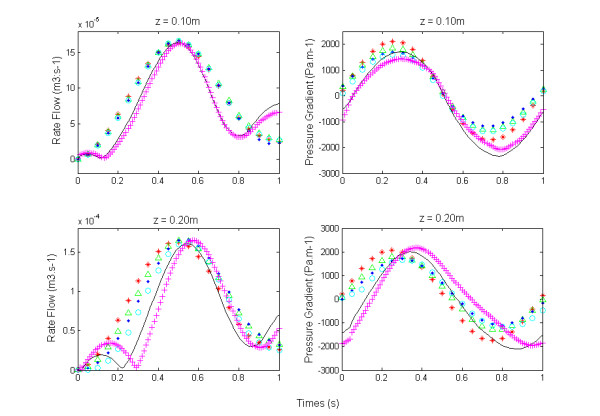
**Signal of rate flow and pressure gradient at various sites.** The pressure gradient and rate flow are computed at z = 0.10m and z = 0.20m.

**Figure 5:**
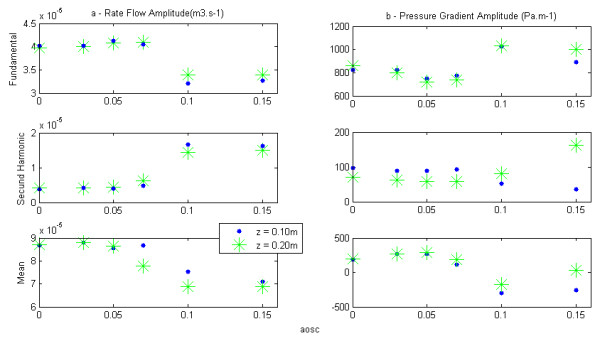
**(a) Amplitude of rate flow – (b) Amplitude of pressure gradient.** The amplitudes are determined from a Fourier transform of respective signals computer in z =0.10m and z = 0.20m.

To examine the influence of distensibility of the arterial wall on the pressure gradient and ra**t**e flow, we have represented in Figure 4(a) and (b)their respective waveforms calculated in each model at 10 cm and 20 cm from the entry. The mean and pulsatile amplitudes of these two quantities are shown in Figure 5. The representation of these amplitudes as a function of aosc allows us to examine the effect of the elasticity of the wall of large arteries on the hemodynamic, locally (mean flow) and at distance (pulsatile flow). In fact, aosc, which by definition characterizes the distensibility of the arteries, also informs us on their elasticity. The evolution of the fundamental amplitude of the longitudinal pressure gradient, $-\frac{\partial P}{\partial z}$ as a function of aosc (Figure 5(b)) presents a minimum at aosc = 0.05, while the mean pressure gradient is maximum (Figure 4(b)).

### Longitudinal impedance

**Figure 6:**
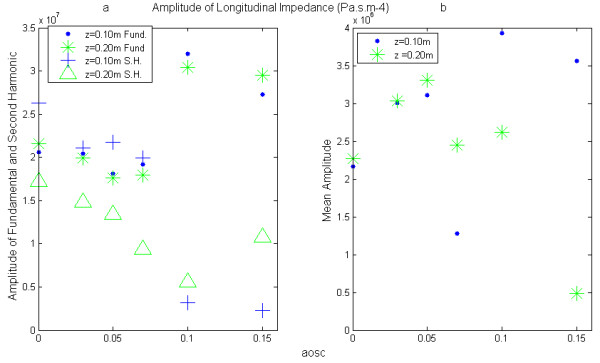
**Amplitude of Longitudinal impedance.** The amplitudes are computed from the ratios of amplitudes of the pressure gradient and of the rate flow.

The longitudinal impedance was obtained by performing the ratio of the amplitude of the pressure gradient and rate flow. As shown in Figure 6(a) the evolution of the fundamental amplitude of the longitudinal impedance as a function of aosc also has a minimum at aosc = 0.05. The value of aosc = 0.05 appears to represent a critical point of the movement of the wall beyond which the fluid - wall interaction would occur in both directions (fluid to wall and wall to fluid).

## Discussion

The Fourier analysis of various pressure pulses obtained at the inlet of the blood vessel, shows that its amplitude decreases with aosc (Figure 2(a)). This indicates the role of damping played by the nonlinear elasticity of the wall. This last result is similar to the experimental results of Armentano [16]. With regard to the wave of rate flow and of the pressure gradient at the inlet and at distance (10cm, 20cm) we found a distortion of their shape that becomes more pronounced when the distensibility of artery increases (see Figures 2(b), 4(a) and (b)). Fourier analysis shows the presence of a second harmonic whose amplitude increases with aosc (Figures 3(a) and 5), despite that the wave of the pressure at the inlet was imposed sinusoidal. The appearance of this second harmonic reflects the fact that the strong coupling fluid–structure causes significant convective acceleration. These quantities give us an answer on the shape of the pressure and of the flow wave-contour in very elastic arteries like the aorta.

The Evolution of the fundamental amplitude of longitudinal pressure gradient, $-\frac{\partial P}{\partial z}$ (Figure 5(b)) for aosc > 0.05 is inconsistent with the conventional results of the fluid mechanics, while that for aosc < 0.05 they are in accordance. The high value of the fundamental amplitude of the pressure gradient obtained for aosc ≥ 0.10 shows the influence of the convective accelerations on the pulsatile flow. We observe two regimes of flow, one, depending on the radius and the other dominated by the elastic behavior, these two behaviors being defined by a critical value of aosc_c_ = 0.05. This critical value would correspond to the limit of the radius variation where the assumption of linearization of Navier - Stokes equations can be applied.

The decreasing part of the mean pressure gradient (Figure 4(b)) can be explained only by the fact that the convective acceleration have a significant effect on the flow of large arteries and therefore cannot be neglected for the large strains (aosc ≥ 0.10). For aosc ≥ 0.10 we observe a reverse pressure gradient. This suggests that the convective effects caused by the elasticity of large arteries play an important role on the hemodynamic of this type of artery by reducing the wall arterial stress. This finding would result in a reduction of energy dissipation and preservation of endothelial cells [23,24]. In summary, we can say that the influence of the convective acceleration developed on the flow is felt locally (mean pressure gradient) and at distance (pulsatile gradient pressure).

In the light of the above remarks, it seems that the nonlinear model preserves the pulse shape much better than the linear propagation mathematical model. The shape of the pressure gradient pulses, obtained from the aosc values equal to 0.10 and 0.15, reproduces with a certain similarity the pressure gradient pulses encountered in large elastic arteries like the aorta. On these physiological curves, one finds the same type of diastolic decrease (except the incisura caused by the closure of the aortic valve) which is traditionally explained by the superposition of the incident wave and reflected wave. However, several experimental studies have been designed to test the accuracy of the theoretical formulations of propagation in describing the physiological phenomenon [12-14]. These experiments have shown that many hemodynamic quantities computed by the linear propagation mathematical models are very different from the corresponding measured values and could not explain the deviation between observations and theory. To get a better response with the linear model, Reuderink and al [25] introduce the wall viscoelasticity and conclude that wall viscoelasticity is able to compensate the lack of convective accelerations in the linear model quite well. In other studies [26] this is the tapering of the tube that is introduced; however, this parameter is not involved in the large arteries. It seems that the nonlinear terms of the Navier–Stokes equations increase with the deformation, meaning that the elasticity of the wall also plays a role in the shape of the pulse decay by making it less steep.

The physical interpretation of the changes in the pressure gradient according to the variation in radius can be performed only when is reported to the rate flow, hence the calculation of the longitudinal impedance. We have therefore calculated the amplitudes of longitudinal impedance from the values of longitudinal pressure gradient and rate flow for each simulation. The fundamental amplitude of the longitudinal impedance Figure 6(a) has also a minimum at aosc = 0.05. The decreases of the impedance for aosc ≤ 0.05 reflects the fact that the flow becomes less resistant due to the increase in radius during the cardiac cycle, whereas, for aosc > 0.05, it tends to increase and to become more important than the rigid case (aosc = 0). The results found for aosc ≤ 0.05 are consistent with those observed in medium-sized arteries, which main role is to perfuse the blood downstream (see additional file 1). This last result is also encountered in large arteries in some pathology leading to a hardening of the arteries such as hypertension or atherosclerosis. We can explain the increase of the fundamental longitudinal impedance, for values of aosc ≥ 0.05 by the fact that a part of the volume of fluid remained stored inside the blood vessel (see additional file 2 and 3). We have seen, above, the importance of convective effects for these values of aosc, which also contributes to mass transport. Normally, the increases of the fundamental pressure gradient amplitude would have led to a significant increase in the fundamental rate flow amplitude. However, we observe the opposite. In fact, we see that the pulsatile rate flow diminishes with aosc at a distance of 20 cm, while it was practically constant at the entrance. These results are very remarkable for values of aosc equal to 0.10 and 0.15, encountered in very elastic arteries. Thus, knowledge of the longitudinal impedance allows us to assess the ability of the artery to store a certain volume of blood.

As shown in Figure 6(b) the mean amplitude of the longitudinal impedance is much smaller than the pulsatile component (fundamental and second harmonic). In fact, different studies [27,28] on the "sleeve effect" and on the hemodynamic effects of vein grafts show that it is the pulsatile components that are most useful to quantify the performance of the local arterial function. However, their studies are based on Womersley's solutions applicable only in small arteries where the parietal displacements (aosc) are well below 0.05. Our focus being to study the role of wall elasticity on the local hemodynamic and chiefly at the level of large arteries, we have not, in our simulation, used restrictive assumptions on the convective acceleration terms which are the nonlinear terms of the Navier - Stokes. In [27,28] the authors have also shown that the longitudinal impedance, increases with the frequency, we observe the same trend for the second harmonic at z = 0.10m and aosc < 0.05, see Figure 5(a). Nevertheless, for the other cases (z = 0.10m–aosc > 0.07 and z = 0.20m–0 ≤ aosc ≤ 0.15) our simulation shows that the second harmonic of the longitudinal impedance is lower than the fundamental and decreases when aosc increases. This is due to the fact that the rate flow and the pressure gradient vary in the same proportion and thus, the convective effects are less important at frequencies above the fundamental. Although the artery longitudinal impedance is usually used as intermediate parameter in calculations, our study has shown a correlation between the longitudinal impedance and the mechanical properties of the wall artery, meaning that it can constitute a clinical important clue for the screening of diseases caused by a change in the mechanical behavior of arteries. Indeed, several studies have shown the incidence of arterial stiffness on cardiovascular disease. Our previous work [29,30] have shown that the pressure gradient and rate flow can be determined from the rate flow and therefore from velocimetry data at the center of blood vessel, thus, it would be interesting to access this index from non-invasive techniques like ultrasonic Doppler or MRI.

In our numerical model we have been subject to certain limitations as the frequency range and reflections. We do not consider multiple reflections caused by the structure of the arterial system because we want to show the response of the elasticity of the arteries on the flow [13,31,32]. Indeed, previous work [30,33-35] shows that the extraction of the incident wave would assess the performance of the heart and arterial system. Regarding frequency, the frequency of 1 Hz is often used in the literature because it represents the fundamental frequency of blood pressure that determines the shape and amplitude of the waveform flow.

## Conclusions

The main results concern the effects of the elasticity and the loss of elasticity of the arterial wall on the flow waveform and the hemodynamic.

The study of the waveform is useful for the hemodynamic signal processing. Our results show that the variation in the radius of the artery during a cardiac cycle effects the hemodynamic. We observe two types of behavior defined by a critical point corresponding to a variation of the radius equal to 5% (aosc). This critical point represents the limit of deformation of the arterial wall beyond which the convective acceleration terms play a significant role on the flow.

The longitudinal impedance was calculated to interpret the effects of convective acceleration on the flow. The results show that for aosc > 0.05 the convective acceleration decelerate the flow so that a portion of blood volume is retained in the upstream of the artery. The longitudinal impedance would, therefore, be a clinically useful parameter to assess the elasticity of the arteries, in order to detect and localize vascular diseases affecting the wall of the elastic arteries like the aorta. Thus, determination of the longitudinal impedance, for a functional exploration of the arterial system, could be performed by non-invasive techniques such as ultrasonic Doppler velocimetry.

## Competing interests

The authors declare that they have no competing interests.

## Authors' contributions

RBS has been involved in criticizing the manuscript and agreed to its publication. Both authors read and approved the final manuscript.

## Supplementary Material

Additional file 1 **Movie simulating the flow when aosc = 0.** This film was made from hemodynamic data obtained in our simulation. This model simulates flow in large arteries (1 cm radius) where the wall does not deform aosc = 0: pathological case. Click here for file

Additional file 2 **Movie simulating the flow when aosc = 0.05.** This film was made from hemodynamic data obtained in our simulations. This model simulates the flow in large arteries (1 cm radius), where the deformation is small compared to that of large arteries AOSC = 0.05: pathological case. Click here for file

Additional file 3 **Movie simulating the flow when aosc = 0.1.** This film was made from hemodynamic data obtained in our simulations. This model simulates the flow in large arteries (1 cm radius), where the deformation is similar to that found in large arteries AOSC = 0.1: normal case. Click here for file
