# Supplementary figures and images for: Influence of the distensibility of large arteries on the longitudinal impedance: application for the development of non-invasive techniques to the diagnosis of arterial diseases
Source: Nonlinear Biomed Phys. 2012 Apr 16;6:2. doi: 10.1186/1753-4631-6-2 (PMC3442960; doi:10.1186/1753-4631-6-2)

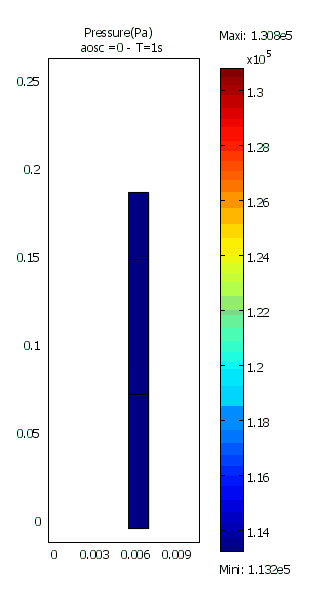

Supplement: Additional file 1 — Movie simulating the flow when aosc = 0. This film was made from hemodynamic data obtained in our simulation. This model simulates flow in large arteries (1 cm radius) where the wall does not deform aosc = 0: pathological case. [file 1753-4631-6-2-S1.gif]

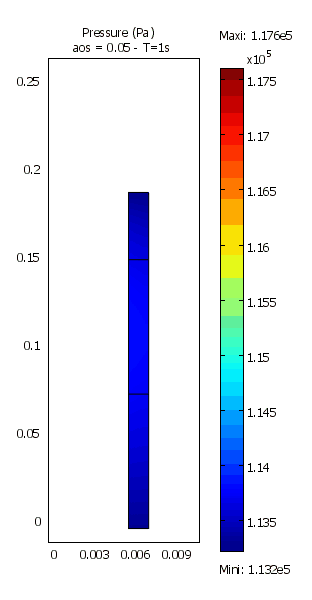

Supplement: Additional file 2 — Movie simulating the flow when aosc = 0.05. This film was made from hemodynamic data obtained in our simulations. This model simulates the flow in large arteries (1 cm radius), where the deformation is small compared to that of large arteries AOSC = 0.05: pathological case. [file 1753-4631-6-2-S2.gif]

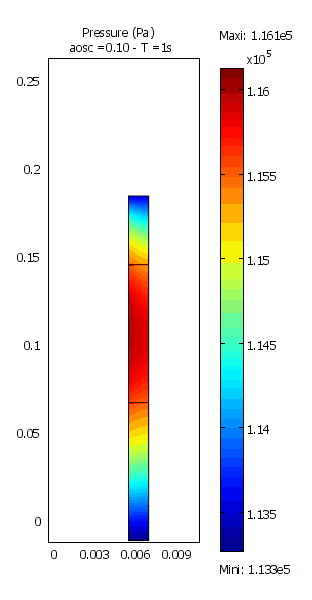

Supplement: Additional file 3 — Movie simulating the flow when aosc = 0.1. This film was made from hemodynamic data obtained in our simulations. This model simulates the flow in large arteries (1 cm radius), where the deformation is similar to that found in large arteries AOSC = 0.1: normal case. [file 1753-4631-6-2-S3.gif]
